# Etude rétrospective de 50 cas de fractures du pilon tibial chez l’adulte dans le Département d’Orthopédie, Centre Hospitalier Universitaire Habib Bourguiba de Sfax, Tunisie

**DOI:** 10.11604/pamj.2021.39.223.28673

**Published:** 2021-08-02

**Authors:** Nizar Sahnoun, Wassim Naiefar, Mohamed Ali Rekik, Bardaa Tarek, Ameur Abid, Hassib Keskes

**Affiliations:** 1 Service d’Orthopédie et de Traumatologie, Centre Hospitalier Universitaire Habib Bourguiba, Sfax, Tunisie

**Keywords:** Pilon tibial, cheville, fracture, traitement chirurgical, Tibial pilon, ankle, fracture, surgical treatment

## Abstract

Les fractures du pilon tibial sont des fractures polymorphes et posent un problème thérapeutique et pronostic .Le but de notre travail est d´étudier le profil épidémiologique de ces fractures, d´évaluer nos résultats sur le plan anatomique et fonctionnel. Nous avons colligé 50 fractures du pilon tibial traitées et suivies entre 2004 et 2013 au Service d´Orthopédie et de Traumatologie de l´hôpital Habib Bourguiba de Sfax. Les résultats fonctionnels ont été évalués par le score Ankle-Hindfoot. Les résultats radiologiques ont évalué la consolidation. Le traitement était par ostéosynthèse interne dans 30 cas, par fixateur externe dans 11 cas et par un traitement combinant les deux techniques dans 9 cas. Au terme de cette étude, nos résultats fonctionnels ont été jugé bons et très bons dans 36 cas. Nous avons noté 30 cas de consolidation, avec 18 cas de cal vicieux et deux cas de pseudarthrose. La prise en charge thérapeutique des fractures du pilon tibial est difficile dans certain cas, une ostéosynthèse solide avec une réduction anatomique est le garant d´un résultat fonctionnel satisfaisant.

## Abstract (en)

Tibial pilon fractures are polymorphic fractures posing therapeutic and prognostic challenges. The purpose of this study is to examine the epidemiological profile of these fractures and to assess our findings from an anatomical and functional point of view. We collected data fom 50 medical records of patients treated for tibial pilon fractures and monitored over the period 2004-2013 at the Orthopedic-Trauma Department of the Habib Bourguiba University Hospital in Sfax. Functional outcomes were assessed using the Ankle-Hindfoot score. Radiological findings were used to determine fracture healing. Treatment was based on internal osteosynthesis in 30 cases, external fixation in 11 cases and on a combination of both techniques in 9 cases. At the end of this study, functional outcomes were good and very good in 36 cases. Thirty cases of fracture healing were reported, with 18 cases of vicious callus and two cases of pseudosteoarthritis. Therapeutic management of tibial pilon fractures is difficult in some cases. Solid osteosynthesis with anatomical reduction is the only therapeutic option to secure a satisfactory functional outcome.

## Introduction

Les fractures du pilon tibial sont des fractures articulaires métaphyso-épiphysaires avec un fort potentiel d´instabilité sagittale. Ce sont des lésions graves, le pronostic fonctionnel est souvent défavorable, le traitement chirurgical représente la meilleure option, dont la réussite est conditionnée par une très bonne reconstitution anatomique de la surface articulaire [1]. Ce type de fracture demeure un problème d´actualité [2]. Ce sont des fractures assez rares [3], et elles sont graves vu leurs complexités leurs difficultés thérapeutiques et l´absence de couverture musculaire avec une vascularisation pauvre rendant le pronostic sévère dominé par le risque de nécrose cutanée, d´infection, de cal vicieux, de pseudarthrose voir de l´arthrose tibio-tarsienne. Malgré l´amélioration de la qualité de prise en charge de ces fractures [4]. Le but de cette étude est de décrire nos résultats fonctionnels et anatomiques des fractures du pilon tibial.

## Méthodes

**Cadre de l'étude:** nous avons colligé 50 fractures du pilon tibial traitées et suivies entre 2004 et 2013 au service d´Orthopédie et de Traumatologie de l´hôpital Habib Bourguiba de Sfax.

**Collecte des données:** la collecte des cas de fractures du pilon tibial s´est faite à partir des registres médicaux. Des fiches d´exploitation préétablies ont été remplies regroupant les paramètres épidémiologiques, cliniques, thérapeutiques et évolutifs, ainsi qu´à la convocation des patients pour évaluer les résultats à long terme.

### Sources de données/mesures

Nous avons recensé l´âge le sexe les antécédents des patient les circonstances de survenu. Tous les patients ont été explorés par une radiographie de la cheville face et profil et par un scanner de la cheville chaque fois que le bilan standard a été jugé insuffisant. Nous avons adopté la classification de l´Association Suisse pour l´étude de l´Ostéosynthèse (AO) qui se base sur le niveau et le trait de fracture, et le degré de comminution métaphysaire. Cette classification distingue trois types: le type A: fracture extra-articulaire (métaphysaire), le type B: fracture articulaire à trait(s) simple(s) réalisant une séparation, sans comminution épiphysaire et le type C: fracture enfoncement articulaire, avec comminution épiphysaire [5]. Le traitement était de de règle chirurgicale par les voies: antéro-interne, antéro-externe, antérieure, interne et postéro-externe selon le type de fracture. Les résultats fonctionnels ont été évalués par le score de l´*American Orthopaedic Foot & Ankle Society* (AOFAS) [6] (Tableau 1): the Ankle-Hind foot Scale, les résultats radiologiques sont jugés par la consolidation ou non en bonne position ou en cal vicieux.

**Tableau 1:** ankle-hind foot scale

1) Douleur	
Aucune	40
Minime, occasionnelle	30
Modérée, quotidienne	20
Sévère, presque toujours présente	0
**2) Fonction**	
a-Limitation des activités:	
Pas de limitation	10
Pas de limitation des activités quotidiennes, limitation des activités de détente	7
Limitation des activités quotidienne et de détente	4
Limitation sévère des activités quotidiennes et de détente avec nécessité d´une aide telles que des cannes, un cadre de marche voire un fauteuil roulant	0
b- distance maximale de marche:	
> 1500 mètres	5
Entre 1000 et 1500 mètres	4
Entre 500 et 1000 mètres	2
<500 mètres	0
c- **Surface de marche:**	
Aucune difficulté quelle que soit la surface	5
Quelques difficultés sur le terrain irrégulier, dans les escaliers, lors de la marche en descente, sur les échelles	3
Sévères difficultés sur les terrains irréguliers, dans les escaliers, lors de la marche en descente, sur les échelles	0
d- **Boiterie:**	
Aucune ou minime	8
Evidente	4
Marquée	0
e- **Mobilité dans le plan sagittal**	
Normal ou limitation minime (30° ou plus)	8
Limitation modérée (15°- 29°)	4
Limitation sévère (< 15°)	0
f- **Mobilité de l´arrière pied (inversion-éversion)**	
Normal ou limitation minime (75% à 100% de la normale)	6
Limitation modérée (25% à 74% de la normale)	3
Limitation sévère (< 25% de la normale)	0
g- Impression subjective de stabilité de la cheville	
Stable	8
Manifestement instable	0
**3) Alignement avant/Arrière pied**	
Bon, pied plantigrade, médio-pied bien aligné	10
Moyen, pied plantigrade, certain degrés de mal alignement du médio-pied, pas de symptômes	5
Mauvais, pied non plantigrade, mal alignement sévère, symptômes	0

**Les variables quantitatives et qualitatives:** les données qualitatives ont été décrites en nombre et en pourcentage. Les données quantitatives ont été décrites par des moyennes et des écart-types.

## Résultats

**Participants:** quarante-quatre (44) des cas étaient de sexe masculin d´âge moyen de 41 ans. Le recul moyen est de 22 mois.

### Données descriptives

**Figure 1:**
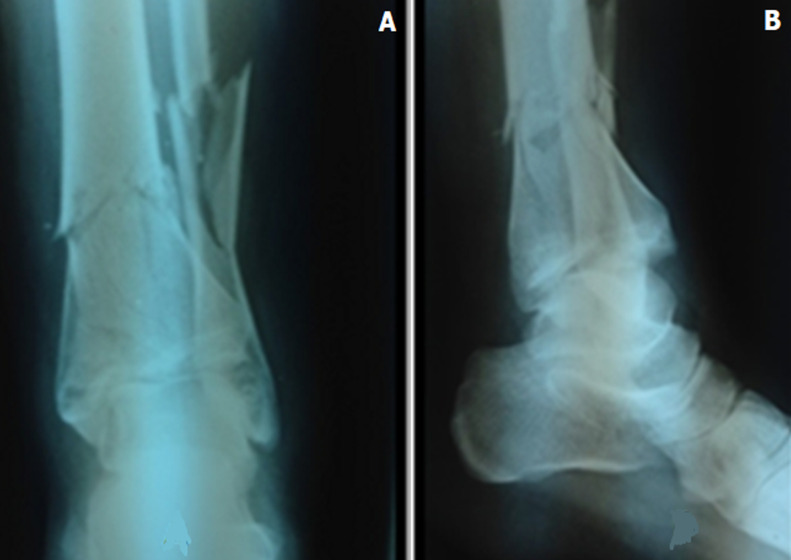
radiographie de la cheville face (A) et profil (B) montrant une fracture du pilon tibial droit Type C3 associé à une fracture de la malléole externe droite

Les circonstances étiologiques étaient dominées par les traumatismes à haute énergie dont les chutes d´un lieu élevé, 24 cas suivis des accidents de la voie publique dans 13 cas. Selon la classification de l´AO on a noté 11 cas de type C3 (Figure 1). Les fractures étaient ouvertes dans 17 cas. Les patients ont été opérés en urgence avec un délai opératoire inférieur à 24h dans 29 cas, de 1 à 7 jours dans 17 cas, et pour 4 cas, ce délai était de plus d´une semaine il s´agissait de patients initialement traités par traction trans-calcanéenne jusqu´à amélioration de l´état cutané puis traités chirurgicalement. Les voies d´abord étaient antéro-interne pour 14 cas, antéro-externe pour 8 cas, antérieure pour 4 cas, postéro-interne pour trois 3 cas et postéro-externe dans un cas.

**Figure 2:**
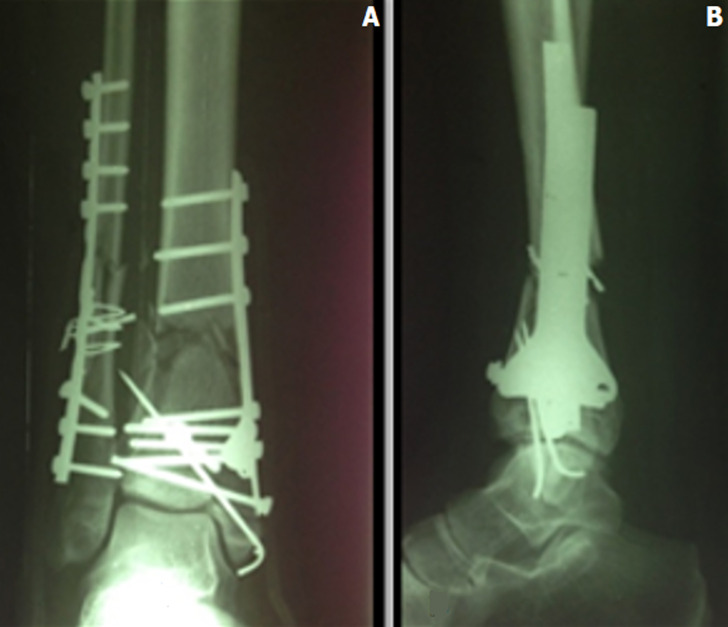
radiographie de la cheville face (A) et profil (B) montrant une synthèse par plaque vissé en trèfle et embrochage avec synthèse de la fibula par plaque vissé 1/3 de tube et cerclage

**Figure 3:**
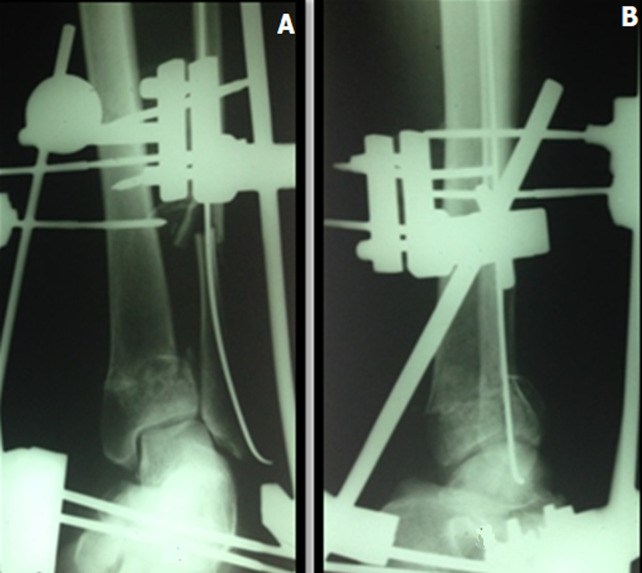
radiographie de la cheville face (A) et profil (B) montrant une synthèse par fixateur externe et embrochage du péroné

Trois types de traitement ont été utilisés: ostéosynthèse interne (Figure 2) dans 30 cas, ostéosynthèse externe (Figure 3) dans 11 cas, et traitement combiné (fixateur externe et synthèse à minima) dans 9 cas. La greffe d´os spongieux autologue, prélevé de la crête iliaque homologue, a été pratiquée dans un seul cas de défect osseux. L´ostéosynthèse de la fibula constitue le premier temps de l´intervention. Elle a été pratiquée dans 22 cas des patients ayant eu une fracture concomitante de la fibula.

**Figure 4:**
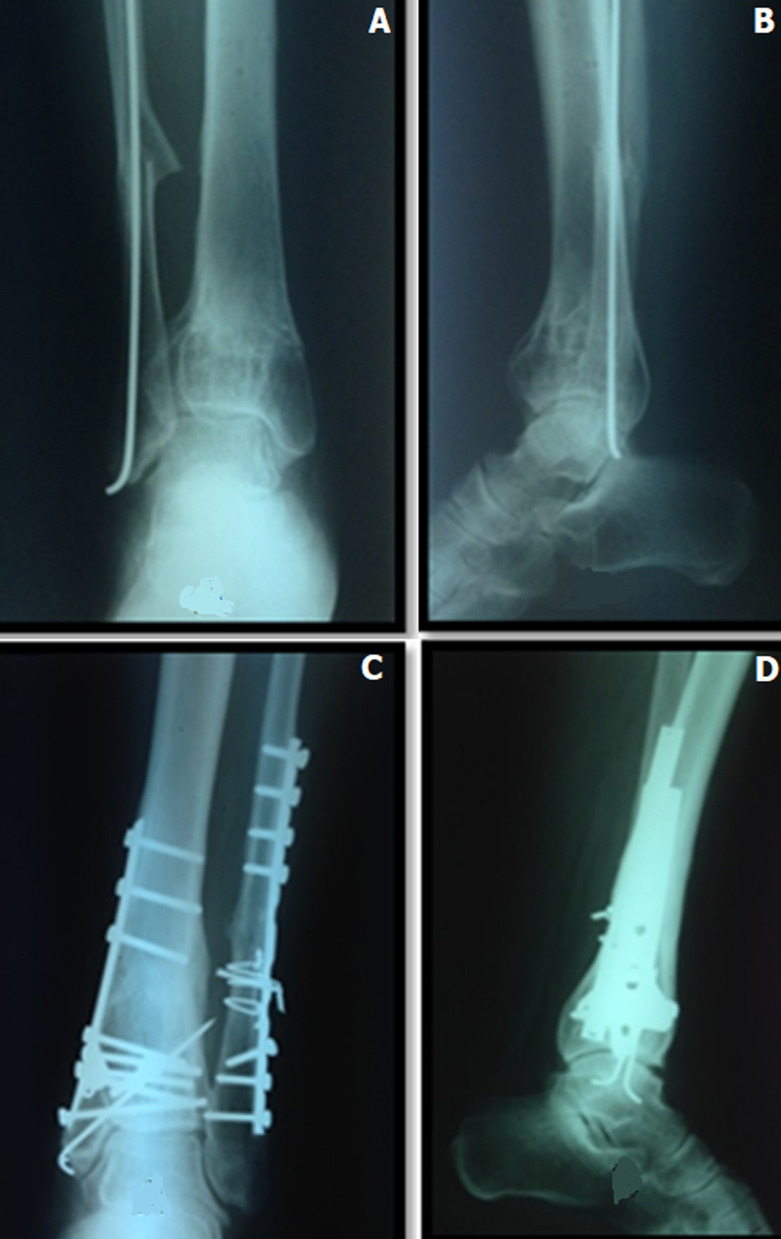
radiographie de la cheville de face (A) et de profil (B) montrant une consolidation de la fracture après fixation externe (C) et synthèse interne (D)

Le délai de consolidation était variable de 8 à 28 semaines (Figure 4). Le délai moyen pour une reprise de l´appui partiel était de 8 semaines. Les complications relevées étaient l´infection dans 6 cas, la nécrose cutanée dans 4 cas, l´algodystrophie dans 7 cas, le sepsis sur matériel dans 7 cas, les cals vicieux dans 18 cas, la pseudarthrose dans 2 cas et l´arthrose dans 17 cas. Les résultats cliniques selon le score “AOFAS Ankle-Hindfoot Scale”, ont été satisfaisants dans 36 cas, moyen dans huit cas et mauvais dans six cas des cas (Tableau 2). Dans notre série, nous avons relevé 2 cas de pseudarthrose et 11 de cals vicieux extra-articulaires et sept de cal vicieux intra-articulaires.

**Tableau 2:** répartition des résultats fonctionnels

Résultats	Effectif	Pourcentage %
**Très Bon**	21	42
**Bon**	15	30
**Moyen**	8	16
**Mauvais**	6	12
**Total**	50	100

## Discussion

Les fractures du pilon tibial sont des traumatismes rares et graves. Elles représentent 1 à 10% des fractures du membre inférieur et 5 à 10% des fractures du tibia selon Calori et Poyanli [7, 8]. Notre population est jeune d´âge moyen de 41 ans, ceci est dû à ce que ces fractures intéressent l´adulte jeune et actif [9, 10]. Le mécanisme le plus fréquent était l´accident de la voie publique ce qui est expliqué par le fait que les fracture du pilon sont la conséquence de traumatismes violents à haute énergie [11]. La tomodensitometrie (TDM) est intéressante dans l´évaluation des fractures complexes du pilon tibial [11, 12]. Nous l´avons demandé chaque fois qu´on a jugé que le bilan standard insuffisant. Il nous a servi dans la planification préopératoire, dans la mesure où elle permet d´identifier le nombre de fragments centraux, l´impaction, la direction du trait de fracture et les fractures non déplacées qui peuvent être passé inaperçues.

La multitude des classifications reflète bien la difficulté de faire part entre le mécanisme lésionnel et les lésions observées, mais deux seulement ont été très utilisée dans la littérature et qui sont les classifications de Rüedi et Algöwer et celle de l´AO. Nous avons adopté la classification de l´AO qui nous parait plus exhaustive. Le traitement des fractures du pilon tibial était chirurgical pour tous nos patients car c´est la seule méthode qui permet de restaurer la congruence articulaire tibio-talienne, garantissant une bonne fonction de la cheville [1]. Notre attitude a été inspirée de celles de Heim [12]. Elles comportent 4 étapes: ostéosynthèse de la fibula pour redonner la longueur de la jambe, réduction anatomique de la surface articulaire tibiale, comblement du défect spongieux par greffe autologue et ostéosynthèse stable du pilon tibial. Nous avons opéré notre patient en urgence différée. D´après Arlettaz *et al*. [13] et Helfet *et al*. [14], il est préférable de différer l´intervention 7 à 10 jours plus tard en attendant l´amélioration de l´état cutané. Dans notre série, le délai moyen entre le traumatisme et l´acte opératoire était de 6,2 jours. Nous avons utilisé différents voies d´abord en effet la voie d´abord des fractures du pilon est discuté dans la littérature [15-17] et sera planifiée après une étude radiologique de la fracture avec ses principaux fragments. Dans la littérature la plupart des auteurs [17, 18] s´accordent sur le fait que la réduction à ciel ouvert permet la meilleure restitution de la forme anatomique du pilon tibial et permet ainsi d´obtenir un meilleur résultat. Après la réduction nous n´avons rencontré un seul cas de defect osseux nécessitant une greffe bien que certains auteurs pensent que le défect obtenu après la reconstruction métaphyso-épiphysaire nécessaire un comblement selon plusieurs auteurs [12, 19, 20] pour son rôle mécanique de support osseux, et son rôle biologique représenté par la stimulation de l´ostéogenèse et donc la consolidation.

Nous avons autorisé l´appui dans un délai moyen de 8 semaines, en effet l'appui est encouragé le plus tôt possible, partiel à partir de de 6 à 8 semaines post opératoire selon Bastias [21], il sera par la suite en charge. Cet appui dépendra aussi du type de fracture et de la qualité de l'ostéosynthèse. Nous avons déploré 6 cas d´infection, cette dernière constitue l´une des principales complications postopératoires. Elle peut être sévère, touchant aussi bien les parties molles que le squelette. Le taux d´infection dans les fractures du pilon tibial est variable dans la littérature. Ce taux est semblable aux taux rapportés par les meilleures séries. Ce taux est expliqué par le respect des parties molles soit par le report de la chirurgie à foyer ouvert après amélioration de l´état cutané, soit l´utilisation du fixateur externe, soit l´utilisation des réductions indirectes avec ostéosynthèse à minima. Dans notre série, la moyenne du score de l'AOFAS était de 85,4 points. Ces résultats sont conformes aux résultats retrouvés dans la littérature. Encinas-Ullan [16] a trouvé un score AOFAS moyen de 84,9 points, Bastias [21] a eu un score moyen de 89 Kao [22] aussi a obtenu un score moyen de 87,3.

Dans notre série, nous avons relevé 2 cas de pseudarthrose. Le taux de pseudarthrose rapporté dans les séries publiées est variable, Bastias [21] n´a pas noté de pseudarthrose en utilisant une ostéosynthèse interne. Par contre Pugh [23] a obtenu un nombre significativement plus élevé de pseudarthroses chez ses patients traités par fixateurs externes en les comparants à ceux traités par synthèse interne. Dans notre série on a trouvé 11 cas de cals vicieux extra articulaires et 7 cas de cal vicieux intra-articulaires. Cette complication a toujours pour origine une erreur thérapeutique, qu´il s´agisse d´un défaut de réduction ou d´une mise en charge trop précoce, mais certains cals vicieux sont pratiquement inévitables après des fractures comminutives du pilon tibial malgré une ostéosynthèse parfaite [16, 21-23]. Certes notre étude a décrit de façon objective les résultats fonctionnels et radiologiques de cette fracture qui pose un réée problème pronostique et thérapeutique mais la cohorte faible de notre population ne nous permet pas de dégager les facteurs de mauvais pronostiques pour ce type de fracture.

## Conclusion

Le traitement chirurgical reste le traitement de choix de ces fractures mais de réalisation difficile, nécessitant un planning pré-opératoire approprié, tenant en considération le type de fracture et l´état cutané. C´est l´ostéosynthèse interne à foyer ouvert qui a donné globalement les meilleurs résultats cliniques, mais le traitement à foyer fermé par fixateur externe, associé à une ostéosynthèse du péroné ou à une ostéosynthèse à minima du tibia, a montré son efficacité réelle et doit avoir sa place particulièrement en cas de comminution importante et de lésions graves.

### Etat des connaissances sur le sujet

- Les fractures du pilon tibial posent un problème thérapeutique;
- Les fractures du pilon tibial posent un problème pronostique à cause des douleurs séquellaires et des troubles trophiques au niveau de la cheville;
- Le traitement des fractures du pilon tibial chez l´adulte est toujours chirurgical.

### Contribution de notre étude à la connaissance

- La compréhension de la fracture est un temps capital dans la planification thérapeutique;
- Une ostéosynthèse solide donne de bons résultats fonctionnels;
- La mauvaise réduction en peropératoire est responsable des cas de cal vicieux.
